# Frequency of Familial Mediterranean Fever Gene Mutation in Patients Presenting With Joint Pain and Diagnosed With Acute Rheumatic Fever

**DOI:** 10.7759/cureus.43001

**Published:** 2023-08-05

**Authors:** Ufuk U Gullu, İsmail Balaban, Soner Sertan Kara, Oğuzhan Yaralı, Ayberk Türkyılmaz, Sevcan İpek, Şeyma D Güllü, Osman F Çalışkan

**Affiliations:** 1 Pediatric Cardiology, Hatay Mustafa Kemal University, Hatay, TUR; 2 Pediatric Cardiology, Yeni Yuzyıl University, Istanbul, TUR; 3 Pediatric Infectious Diseases, Adnan Menderes University, Aydın, TUR; 4 Medical Genetics, Erzurum Regional Training and Research Hospital, Erzurum, TUR; 5 Pediatrics, Hatay Mustafa Kemal University, Hatay, TUR; 6 Pediatric Medicine, Hatay Mustafa Kemal University, Hatay, TUR

**Keywords:** yalcinkaya-ozen criteria, mefv gene mutation, familial mediterranean fever, joint pain, acute rheumatic fever

## Abstract

Introduction

Acute rheumatic fever (ARF) is a non-suppurative systemic inflammatory disease that manifests 1-5 weeks following a Group A beta-hemolytic streptococcal infection. On the other hand, familial Mediterranean fever (FMF) is a hereditary autoinflammatory disease characterized as an autosomal recessive disease, with affected individuals having pathogenic mutations in the Mediterranean fever gene *(MEFV)* gene located on the short arm of chromosome 16. FMF and ARF have overlapping symptoms and signs, and both disorders are common in Turkey. In ARF, the target organ is the heart, while in FMF, the target organ is the kidney; both organs can benefit from prophylactic measures. Our study aims to determine the frequency of the FMF gene mutation in patients with ARF in Turkey and detect any overlapping conditions.

Method

Patients who were diagnosed with a first-attack ARF between May 2015 and May 2018 were retrospectively screened. Patients who underwent *MEFV* gene analysis considering FMF in the differential diagnosis were included in the study.

Results

In this study, no statistical difference was found between the presence of *MEFV* gene mutations, carditis, high anti-streptolysin-O antibody (ASO) levels, and the groups with monoarthritis, polyarthritis, and polyarthralgia (p >0.05).

Conclusions

In conclusion, patients with ARF should be evaluated for FMF to avoid irreversible complications.

## Introduction

Acute rheumatic fever (ARF) is a non-suppurative systemic inflammatory disease seen 1-5 weeks after a Group A beta-hemolytic streptococcal infection. ARF is diagnosed based on the Jones Criteria, revised by the American Heart Association in 2015. These criteria recommend different diagnostic approaches for low and medium/high-risk populations [1], with Turkey falling into the medium/high-risk group [2]. Familial Mediterranean fever (FMF) is a hereditary autoinflammatory disorder characterized by serosal inflammation (peritonitis, pleuritis, and arthritis) and recurrent fever attacks. The most significant complications include amyloidosis, chronic renal failure, infertility, and chronic arthritis. FMF predominantly affects individuals of Turkish, Armenian, North African, Arabic, and Jewish origins [3]. FMF is an autosomal recessive disease, and affected individuals have biallelic pathogenic mutations in the *MEFV* gene located on the short arm of chromosome 16 (16p13.3). Five mutations, V726A, M694V, M694I, M680I, and E148Q, are prevalent in approximately 75% of Armenian, Arabic, Jewish, and Turkish patients with FMF [4,5]. However, about 10-20% of patients meeting the FMF diagnostic criteria do not have an *MEFV* mutation [6]. FMF and ARF share overlapping symptoms and findings. Both diseases are prevalent in Turkey, making it crucial to distinguish between them for accurate disease management. The current literature lacks studies directly exploring the overlapping or distinctive conditions of ARF and FMF. Therefore, our study aims to determine the frequency of FMF gene mutations in patients with ARF in Turkey and detect overlapping conditions.

## Materials and methods

Hospital records of the patients admitted with complaints of joint pain and diagnosed with their first episode of ARF between May 2015 and May 2018 were evaluated retrospectively. Patients with suspected FMF in the differential diagnosis and whose MEFV gene mutation was tested were included in the study. The diagnosis of ARF was made based on the Jones Criteria revised in 2015. For ARF, the major criteria in medium to high-risk classification are carditis, joint involvement (monoarthritis, polyarthritis, and/or polyarthralgia), Sydenham’s chorea, erythema marginatum, and subcutaneous nodules. Minor criteria are ≥38 °C, PR prolongation, erythrocyte sedimentation rates (ESR) ≥ 30 mm, monoarthralgia, and/or C-reactive protein (CRP) ≥ 3.0 mg/dl [1,2]. All patients were physically examined and evaluated using electrocardiogram and echocardiography in the pediatric cardiology clinic. ESR, serum C-RP levels, and anti-streptolysin O (ASO) levels of the patients were recorded. Genetic analysis was performed regarding FMF from patients diagnosed with ARF and whose clinical findings matched the Yalcinkaya-Ozen criteria. There should be two or more criteria of fever exceeding 38 °C, arthritis, chest pain, abdominal pain, and family history of FMF. At least three attacks should occur and last 6-72 hours. The study was approved by the Clinical Research Ethics Committee of the Health Sciences University Erzurum Regional Training and Research Hospital (No: 2018/12-112).

Gene analysis

Peripheral venous blood (400 μL) was withdrawn into ethylenediamine tetra-acetic acid (EDTA) tubes. Then, genomic DNA was extracted from the peripheral venous blood using the QIAamp® DNA Mini Kit (Qiagen GmbH, Hilden, Germany). All coding exons and exon-intron boundaries of the MEFV gene were sequenced on the Illumina MiSeq Platform using the Agilent SureSelect V2 kit (Agilent, Santa Clara, CA, USA). The raw data were analyzed through the Sophia DDM® data analysis platform. Alignment and variant detection was performed by Pepper®, a basic proprietary algorithm of Sophia Genetics, according to the human genome reference hg19.

Patients with MEFV gene mutations were divided into three groups, those with heterozygous, compound heterozygous, and homozygous genotypes.

Statistical analyses

Statistical analyses were performed using the SPSS 25.0 (IBM Corp., Armonk, NY, USA) package program. Normality analysis was done with the Kolmogorov-Smirnov test for continuous variables. Categorical variables were expressed as frequency and percentage, while continuous data showing a non-normal distribution were expressed as median and interquartile range. Continuous variables that were not normally distributed were analyzed with the Kruskal-Wallis test, and the Chi-square test was used to analyze categorical variables. The statistical significance was taken as p <0.05.

## Results

A total of 56 children aged between 5 and 18 years (median 11.3; IQR 9.8-13.5), of whom 22 (39%) were females and 34 (61%) were males, were included in the study. Eleven patients (20%) had monoarthritis, 38 (68%) had polyarthritis, and 7 (12%) had polyarthralgia. Table 1 shows the patients' demographic characteristics and clinical and laboratory findings by joint involvement. There were no statistically significant differences among the types of joint involvement by age, gender, length of hospital stay, acute phase reactants (ESR and CRP), ASO, fever, cardiac involvement, and MEFV gene mutation rates (p> 0.05). None of our patients had Sydenham's chorea, subcutaneous nodules, and erythema marginatum.

**Table 1:** Demographic, laboratory, and clinical findings according to joint involvement. ^a^ Chi-square, ^b ^Kruskal-Wallis. ESR: Erythrocyte sedimentation rate; CRP: C-reactive protein; ASO: Anti-streptolysin O.

	Monoarthritis	Polyarthritis	Polyarthralgia	P-value
Sex n (%)				0.27^a^
Female	2 (3.6)	17 (30.4)	3 (4)	
Male	9 (16.1)	21 (37.5)	4 (7)	
Age (year) (median; IQR)	11.6; 5.6	11.2; 3.3	11.9; 2.9	0.9^b^
Day of hospitalization (median; IQR)	14; 11	14; 12	13; 2	0.77^b^
ESR (median; IQR)	42; 37	42; 18	43; 33	0.45^b^
CRP (median; IQR)	6.3; 5	7; 7	5.3; 10	0.89^b^
ASO (median; IQR)	1060; 792	1375; 827	836; 471	0.28^b^
Fever				0.78^a^
No	9 (16.1)	34 (60.7)	6 (10.7)	
Yes	2 (3.6)	4 (7.1)	1 (1.8)	
Aortic Regurgitation				0.89^b^
None	3 (5.4)	9 (16.1)	2 (3.6)	
Mild	5 (8.9)	17 (30.4)	4 (7.1)	
Moderate	0	1 (1.8)	0	
Severe	0	0	0	
Trace	3 (5.4)	11 (19.6)	1 (1.8)	
Mitral Regurgitation				0.32^b^
None	1 (1.8)	1 (1.8)	0	
Mild	7 (12.5)	20 (35.7)	6 (10.7)	
Moderate	0	4 (7.1)	0	
Severe	0	4 (7.1)	1 (1.8)	
Trace	3 (5.4)	9 (16.1)	0	
Carditis Degree				0.08^b^
None	1 (1.8)	0	0	
Mild	10 (17.9)	29 (51.8)	6 (10.7)	
Moderate	0	5 (8.9)	0	
Severe	0	4 (7.1)	1 (1.8)	
MEFV Gene Mutations	7 (12.5)	17 (30.5)	4 (7)	0.5^a^

*MEFV *mutations in ARF patients

At least one heterozygous mutation was detected in 28 out of 56 patients (50%). The detected mutations were M694V homozygous and R202Q homozygous (one patient), E148Q homozygous (one patient), compound heterozygous (nine patients), and single heterozygous mutation (17 patients). The most frequent mutations in the patients were R202Q (37.5%), V726A (7.1%), E148Q (5.3%), I591T (3.6%), and M694V (3.6%). The clinical findings of the 11 patients with compound heterozygous and homozygous mutations are shown in Table 2.

**Table 2:** Clinical findings of patients with heterozygous, compound heterozygous, and homozygous mutations. ESR: Erythrocyte sedimentation rate; CRP: C-reactive protein; ASO: Anti-streptolysin O.

Patient number	Mutation	Joint involvement	Carditis degree	Minor findings
1	M694V Homozygous; R202Q Homozygous	Monoarthritis	Mild carditis	ESR, CRP
2	E148Q Homozygous	Polyarthritis	Severe carditis	ESR, CRP
3	R202Q Heterozygous; V726A Heterozygous	Polyarthritis	Mild carditis	ESR, CRP, PR prolongation
4	R202Q Heterozygous; M694V Heterozygous; V726A Heterozygous	Polyarthralgia	Mild carditis	ESR, CRP, fever
5	P369S Heterozygous; R408Q Heterozygous	Polyarthritis	Mild carditis	ESR, CRP
6	R202Q Heterozygous; V726A Heterozygous	Polyarthritis	Mild carditis	ESR, CRP
7	R202Q Heterozygous; S179I Heterozygous	Polyarthralgia	Mild carditis	ESR, CRP
8	R202Q Heterozygous; I591T Heterozygous	Monoarthritis	Mild carditis	ESR, CRP
9	R202Q Heterozygous; I591T Heterozygous	Polyarthritis	Mild carditis	ESR, CRP
10	R202Q Heterozygous; M680I Heterozygous	Monoarthritis	Mild carditis	ESR, CRP
11	R202Q Heterozygous; K695R Heterozygous	Polyarthritis	Mild carditis	ESR, CRP
12	R202Q Heterozygous	Polyarthritis	Mild carditis	ESR, CRP
13	R202Q Heterozygous	Polyarthritis	Moderate carditis	ESR, CRP, PR prolongation, fever
14	R202Q Heterozygous	Polyarthritis	Mild carditis	ESR, ASO
15	R202Q Heterozygous	Monoarthritis	Mild carditis	ESR, CRP, ASO, fever
16	R202Q Heterozygous	Polyarthritis	Severe carditis	ESR, CRP, ASO,PR prolongation
17	R202Q Heterozygous	Polyarthritis	Moderate carditis	ESR, CRP, ASO
18	R202Q Heterozygous	Polyarthritis	Mild carditis	ESR, CRP, ASO, PR prolongation
19	R202Q Heterozygous	Polyarthritis	Mild carditis	ESR, CRP, ASO
20	R202Q Heterozygous	Monoarthritis	Mild carditis	CRP, ASO, PR prolongation
21	R202Q Heterozygous	Polyarthritis	Mild carditis	ESR, CRP, ASO
22	R202Q Heterozygous	Polyarthritis	Mild carditis	ESR, ASO, PR prolongation
23	R202Q Heterozygous	Polyarthritis	Mild carditis	ESR, CRP, ASO
24	E148Q Heterozygous	Monoarthritis	Mild carditis	ESR, CRP, ASO
25	E148Q Heterozygous	Polyarthritis	Mild carditis	ESR, CRP, ASO
26	V726V Heterozygous	Polyarthritis	Mild carditis	ESR, ASO, PR prolongation
27	V726A Heterozygous	Monoarthritis	Mild carditis	ESR, ASO
28	S339F Heterozygous	Polyarthritis	Mild carditis	ESR, CRP, ASO, PR prolongation

## Discussion

**Figure 1:**
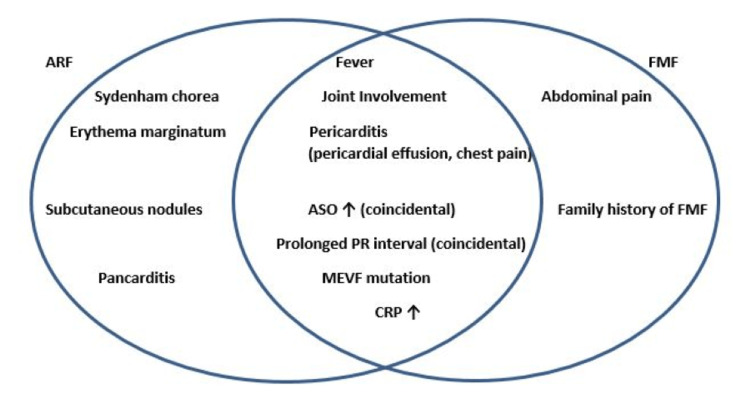
ARF and FMF criteria and/or symptoms coincide with each other. This figure was created by the authors. ARF: Acute rheumatic fever; FMF: Familial Mediterranean fever, ASO: Anti-streptolysin O; CRP: C-reactive protein.

In this study, no statistical difference was found among monoarthritis, polyarthritis, and polyarthralgia groups with MEFV gene mutations and high ASO levels. There was also no significant difference among these groups by carditis. The increase in ASO titer, which indicates that the streptococcal infection has passed, is also a supportive finding [1,2]. Accordingly, one major and two minor or two major criteria must be present in the first attack, which is one of the Jones criteria. Although Sydenham's chorea, subcutaneous nodules, and erythema marginatum are major criteria for diagnosing ARF, none of our patients had these conditions. We diagnosed the first attack according to the modified Jones criteria [1]. FMF attacks also progress with sedimentation and CRP elevation, and ASO elevation is a common laboratory parameter in childhood [7]. In the study, it was concluded that the criteria and/or symptoms of ARF and FMF overlapped with each other (Figure 1).

Secondary prophylaxis with penicillin G benzathine is performed to prevent heart disease in patients with ARF [1,8]. Renal involvement in FMF is the main cause of morbidity, and lifelong colchicine should be used in order to avoid this [9]. The diagnosis of FMF is made based on family history and clinical symptoms. Genetic tests serve to support the diagnosis of FMF [10]. FMF is diagnosed based on the Yalcinkaya-Ozen criteria in Turkey [11]. Genetic tests are used to support FMF diagnosis and exclude other autoinflammatory diseases that mimic FMF [12]. The diagnosis is confirmed upon detecting two pathogenic mutations in the MEFV gene in FMF, which is generally autosomal recessive transitive [11]. The MEFV gene is located on chromosome 16p13.3 and has a 2346 bp coding sequence spanning 10 exons. The MEFV gene produces a 781 amino acid protein known as pyrin, and the most frequent mutations are M694V, E148Q, M680I, and V726A [13]. The clinical severities and penetrance of these mutations are different from each other. For example, the M694V mutation causes the most severe clinical phenotype and is associated with AA-type amyloidosis. The E148Q mutation is associated with reduced penetrance and a mild phenotype. Some E148Q homozygous individuals have been reported to be asymptomatic [14]. V726A homozygotes are also known to show mild phenotypes [15]. However, only one mutation is detected in approximately 33% of patients meeting FMF clinical criteria [6,16]. Furthermore, 10-20% of patients do not carry any mutations [17,18]. Phenotypic expression of FMF has been reported in a significant proportion of patients with only one MEFV mutation [19]. In Turkey, FMF prevalence often shows a substantial regional variance at rates from 1/1000 to 8.8/1000 [20,21]. The MEFV gene mutation rate is 20% among Turkish people [22]. Orün UA et al. found the ARF frequency was 37, 60, and 21/100,000 in Turkey for three 10-year periods [23]. In our study, M694V homozygous and R202Q homozygous were detected in a patient diagnosed with their first attack of ARF. Therefore, we recommend patients with homozygous and compound heterozygous MEFV mutations undergo a pediatric rheumatology examination to evaluate FMF.

In addition, pericarditis is frequent in patients with FMF who are admitted with chest pain complaints [24-26]. ARF can also cause pancarditis; patients may be admitted to the clinic with chest pain complaints [27]. Arthritis occurs in 70-75% of patients with FMF, and acute monoarthritis, which involves large joints, is the most common sign and maybe migratory in some cases [15]. Children with ARF often visit the hospital complaining of arthralgia and/or arthritis signs. The clinical findings of FMF patients may meet the Jones criteria for diagnosing ARF. In addition, an FMF patient with coincidental and/or previous coincidental rheumatic heart disease can easily be diagnosed as the first episode of ARF. Additionally, a high ASO level is a common condition in a healthy population but may show an excessive increase coincidentally during an FMF attack; thus, it may incorrectly be considered evidence of a beta-hemolytic streptococcal infection. The prolonged PR interval is nonspecific and can be seen among healthy individuals [28-29]. It can also be encountered coincidentally in patients with FMF. Tekin M et al. reported that clinical and laboratory findings of ARF overlapped in 5.5% of patients with FMF [30]. Because the diagnostic criteria of ARF and FMF overlap, the clinician may be in a difficult situation regarding overdiagnosis/misdiagnosis. 

The study's limitations are that it is a retrospective single-center study, and the number of patients is small. In addition, another limitation is the lack of long-term follow-up. The strength of the study lies in the performance of genetic tests for FMF in patients with ARF, as misdiagnosis can lead to irreversible complications.

## Conclusions

In this retrospective study, children who were diagnosed with first-episode ARF and who underwent gene analysis for FMF were evaluated. Accordingly, it has been suggested that FMF is a critical disease that should be considered in the differential diagnosis of ARF in patients with joint pain. A differential diagnosis is essential since the morbidity rates of both diseases are relatively high. Patients with ARF should be subjected to FMF evaluation to avoid irreversible complications. Finally, further research is needed to reveal an in-depth understanding of this issue.
